# Biomaterials in Restorative Dentistry and Endodontics

**DOI:** 10.3390/jfb17010017

**Published:** 2025-12-26

**Authors:** Paulo J. Palma, Fabio D. Nascimento

**Affiliations:** 1 Institute of Endodontics, Faculty of Medicine, University of Coimbra, 3000-075 Coimbra, Portugal; ppalma@uc.pt; 2 Center for Innovation and Research in Oral Sciences (CIROS), Faculty of Medicine, University of Coimbra, 3000-075 Coimbra, Portugal; 3 Molecular Biology Division, Federal University of São Paulo, São Paulo 04044-020, Brazil

## 1. Introduction

Dental biomaterials are key innovations in dentistry, serving as essential tools for restoring, preserving, and re-establishing oral health. These materials consist of a diverse range of substances, including polymers, ceramics, metals, and bioceramics, all of which are designed to interact optimally with the oral environment [1]. This research field not only focuses on achieving biocompatibility but also aims to provide both functional and esthetic results. The continuous advancement of dental biomaterials reflects a commitment to improving clinical efficacy, maximizing patient comfort, and ensuring the long-term stability of treatments. By utilizing the latest developments in materials science, bioengineering, and digital technology, we are transforming patient care and setting new standards for oral health.

In recent decades, continuous research and innovation in materials science have deeply transformed dental practice. Today’s dental clinicians rely on a diverse range of biomaterials designed for specific therapeutic purposes, including restorative, prosthetic, regenerative, and endodontic applications. Modified dental composites [2] and bioceramics [3] have become fundamental components of dental practice, offering excellent mechanical properties and chemical stability. Additionally, the use of advanced fabrication technologies, such as computer-aided design and computer-aided manufacturing (CAD/CAM), 3D printing, and digital scanning, has greatly improved precision, reproducibility, and clinical efficiency.

While restorative materials have traditionally been at the forefront of dental innovation, there have also been significant advancements in endodontics, particularly with the role of biomaterials in influencing treatment outcomes. The ideal endodontic material should not only create a strong seal to prevent bacterial microleakage but also demonstrate bioactivity, biocompatibility, and stability within the moist and dynamic conditions of the root canal system [4]. In recent years, bioceramic-based endodontic materials, such as hydraulic calcium silicate-based cements (including mineral trioxide aggregate (MTA) and Biodentine, resin-based sealers, and bioceramic sealers), have transformed the field of endodontics. These materials facilitate the release of calcium and hydroxyl ions, creating an alkaline environment that promotes hard tissue formation and inhibits bacterial growth [5]. Their excellent sealing capability and ability to induce the deposition of hydroxyapatite at the dentin interface make them invaluable for various endodontic procedures, including root end fillings, perforation repairs, pulp capping, apexification, and regenerative endodontic therapies [6]. Furthermore, the bioactive properties of these materials stimulate the regeneration of the dentin–pulp complex, promoting a shift from traditional obturation methods to biologically active systems aimed at preserving pulp vitality and maintaining natural tooth function [7].

The integration of bioactive materials and regenerative principles into endodontics represents a significant shift in approach. Modern regenerative endodontic therapies (RETs) utilize scaffolds, growth factors, and stem cells alongside biomaterials designed to support cellular adhesion, proliferation, and differentiation [8]. These materials serve not only as physical barriers or fillers but also as biological mediators that promote healing and tissue regeneration. Advances in nanotechnology have further enhanced the capabilities of endodontic biomaterials, leading to the development of nanostructured sealers, antimicrobial coatings, and controlled-release systems. These innovations improve disinfection, enhance sealing ability, and modulate the host response.

Combined, these advancements have emphasized the essential role of dental biomaterials as both restorative and regenerative agents. They have transitioned from being mere fillers or structural supports to becoming dynamic, biologically responsive systems that can interact with cells and tissues at the molecular level. This transformation illustrates the profound connection between materials science, cellular biology, and clinical innovation.

In this Special Issue, we explore significant advancements and applications of modern dental biomaterials, highlighting their essential role in restorative, regenerative, and endodontic treatments.

The Special Issue ‘Biomaterials in Restorative Dentistry and Endodontics’ presents eight scientific contributions, submitted by scholars with renowned backgrounds in developing and testing new biomaterials for multiple clinical purposes; it includes one review and seven original research articles.

## 2. Special Issue Highlights

Landmayer et al. [9] assessed the effectiveness of a resin infiltrant (Icon^®^) that had been functionalized with methacrylate epigallocatechin-3-gallate (EGCG) in improving the esthetic appearance and penetration depth (PD) of white spot lesions (WSLs) on enamel. Results showed no significant difference in PD among the three resin groups: infiltrant, infiltrant–EGCG, and infiltrant–EGCG–methacrylate. In terms of color change, infiltrant and infiltrant–EGCG–methacrylate demonstrated similar masking effects to untreated enamel, while infiltrant–EGCG showed intermediate performance. The study concludes that methacrylated EGCG enhances the esthetic masking of WSLs while maintaining effective resin penetration, making it a promising modification for dental resin infiltrants.

The study conducted by Behlau et al. [10] aimed to evaluate the surface roughness of five amalgam-replacement materials, based on glass ionomer cements, over time and in relation to the finishing method used. A total of 960 samples were prepared using standardized 3D-printed molds, and the surface roughness was measured at three time points using a non-contact profilometer: immediately after finishing, after 30 days in distilled water, and after 5000 thermocycles. The study highlights the importance of tailoring finishing techniques to the specific restorative material to optimize surface smoothness and long-term performance, especially in light of regulatory shifts away from amalgam use.

Benetti et al. [10] performed a study that explored the biological performance of two experimental dental pastes, 45S5 bioglass and Bio-C Temp, compared with calcium hydroxide (Ca(OH)_2_) paste. The investigation focused on cytotoxicity, biocompatibility, calcium deposition, and collagen maturation, using both in vitro and in vivo models. In the in vitro tests, dental pulp cells were exposed to material extracts at 1:2 and 1:4 dilutions for durations of 24, 48, and 72 h. Cell viability was assessed using MTT assays and live/dead assays. For the in vivo tests, polyethylene tubes filled with the pastes (or left empty as controls) were implanted in rats. The tissues were analyzed after 7 and 30 days. The authors concluded that the 45S5 bioglass paste showed promising results in promoting continuous collagen formation, supporting its potential as a novel biomaterial in dental applications.

In another interesting study, Dos Santos et al. [11] verified the regenerative potential of poly(aspartic acid) (pAsp) as an alternative pulp-capping agent to mineral trioxide aggregate (MTA) by targeting dentinogenesis signaling pathways. Using an animal model, mechanical pulp exposures were performed on 56 Wistar rats, divided into four groups: control (no treatment), MTA, pAsp, and MTA+pAsp. Treatments were applied directly to exposed pulps, and animals were euthanized after 7 or 21 days for histological and immunohistochemical analysis. The study concluded that pAsp demonstrated effective dentin regeneration without the need for an external calcium source and may serve as a promising alternative to MTA in pulpal therapy.

Naeem et al. [12] evaluated the impact of propolis, a natural antimicrobial extract, on the microhardness of root dentine compared with calcium hydroxide (CH), a widely used intracanal medicament that is known to reduce dentine hardness. Ninety root disks were randomly assigned to three groups: CH, propolis, and a control. Microhardness was measured using a Vickers hardness tester at 24 h, 3 days, and 7 days. The authors concluded that propolis not only avoids the weakening effect of CH on root dentine but also enhances dentine microhardness over time, suggesting its potential as a beneficial alternative intracanal medicament in endodontic therapy.

The manuscript by Herrero-Climent et al. [13] evaluated the horizontal marginal fit of dental prosthesis suprastructures produced using three different techniques: CAD-CAM, laser sintering, and traditional casting. A total of 70 chromium–cobalt (CrCo) models were designed—30 via CAD-CAM, 30 via laser sintering, and 10 via casting—by six different manufacturers using a standardized model. The study concluded that CAD-CAM technology offers superior marginal fit for dental prostheses compared with laser sintering and casting. These findings support the continued adoption of digital production methods in modern prosthodontics.

The first research manuscript published in this Special Issue came from Professor Anuradha Prakki’s group from the University of Toronto [14]. The study investigated the cytotoxicity and antimicrobial properties of poly(N-vinylcaprolactam) (PNVCL) hydrogels loaded with flavonoids, ampelopsin (AMP), isoquercitrin, and rutin as potential intracanal medications for endodontic therapy. The antimicrobial activity of these compounds was tested against five key endodontic pathogens using the microdilution method. Interestingly, the authors concluded that PNVCL hydrogels containing AMP demonstrated both cytocompatibility and potent antimicrobial effects, indicating their potential as effective intracanal medications in endodontic treatment.

Finally, our Special Issue features a systematic review article, authored by Agarwal et al. [15], that evaluated the impact of various irrigating solutions, their combinations, and their activation modes on dentin microhardness in endodontic treatment. The authors concluded that the use of irrigating solutions during endodontic treatment significantly affects the microhardness of root canal dentin. Among the various solutions analyzed, sodium hypochlorite (NaOCl), particularly at a concentration of 2.5% with prolonged contact time, caused the greatest reduction in dentin microhardness. Other agents, such as EDTA, citric acid, glycolic acid, phytic acid, and herbal irrigants, also contributed to dentin softening, while distilled water showed minimal impact. These findings underscore the importance of carefully selecting and timing irrigant application to preserve dentin integrity and optimize the long-term success of endodontic therapy.

## 3. Concluding Remarks

This Special Issue brings together a systematic review and seven research articles that present the latest advancements in biomaterials for restorative dentistry and endodontics. These contributions describe an array of innovative biomaterials, both natural and synthetic, that have been specifically designed for the effective treatment of enamel, dentin, and the root canal. The exceptional quality and rich diversity of the research underscore the critical significance of this field, as well as the cutting-edge developments that are currently shaping it. As we witness the evolution of dental biomaterials, we are not just imagining a future of dentistry; we are actively forging a path toward healthier, more functional, and beautifully harmonious smiles for generations to come.

## Data Availability

No new data were created or analyzed in this study. Data sharing is not applicable to this article.
